# The Effects of Service-Learning on the Civic Attitudes and Self Efficacys of Women University Students in Korea

**Published:** 2020-02

**Authors:** Sook-Young RYU, Wi-Young SO

**Affiliations:** 1. Institute of General Education, Seoul Women’s University, Seoul, Korea; 2. Sports and Health Care Major, Korea National University of Transportation, Chungju-si, Korea

## Dear Editor-in-Chief

In college education, it is necessary to participate in social problem solving as well as focusing on academics and creating learning to bring valuable change for the benefit of individuals and society (1).

Service Learning (S-L) is a teaching and learning method to deepen classroom learning through community service or civic engagement (2). Besides collaborations and partnerships between universities and the community, feedback and reflections connecting specific educational goals with meaningful community service are the most essential for in-context learning in S-L. The reflections are usually focused on the service experience and the demonstration of skills and knowledge acquired (2).

S-L is an effective educational method, especially for self-efficacy (3) and citizenship (4), but the results of academic development through S-L are mixed. That is, students who voluntarily participate in S-L are usually more active, and therefore their academic achievement is also high (5). However, the changes in personality from S-L may not be adequately assessed through methods typically used to evaluate academic development at universities (6). Therefore, the purpose of this study was to investigate the effects of S-L on civic attitudes and the self-efficacy of students, especially female students who will be productive members of Korean society.

The study was conducted on 1,464 female students from Seoul Women’s University, Seoul, Republic of Korea in 2015. The S-L education participation group was 1,184 students (aged 19– 22 yr) and the control group was 298 students (aged 19–22 years).

They were informed of the purpose and process of this study and signed the consent form.

The S-L course participants needed to spend 32 h performing S-L activities, broken down to two hours per week for sixteen weeks; this also included eight hours of orientation and an introspection conference. The control group was asked not to participate in any S-L program for the same semester. The parameters of civic attitudes and self-efficacy were measured before and after intervention through the 16 weeks.

“Civic attitudes” is defined as the responsibility to help others and solve societal problems. The civic attitudes scale was developed by Mabry (7). Self-efficacy is a generalized expectation of personal mastery across a variety of situations. The self-efficacy scale was developed by Sherer et al. (8).

All analyses were performed using SPSS ver. 18.0 (Chicago, IL, USA). All data are presented as mean ± standard deviation. An analysis of the data was performed using the 2 × 2 (group × time) repeated measures analysis of variance. Statistical significance was set at *P*<0.05.

There were interaction effects (time × group) on civic attitudes (positively) (*P*<0.001) and self-efficacy (negatively) (*P*<0.001) (Table 1). The students from the S-L class were able to participate in processes to solve social problems, meet and cooperate with people in the field, and develop an interest in society as well as an awareness of participation through S-L.

**Table 1:** Changes in civic attitudes and self-efficacy after 16 weeks of the Service-Learning program

***Variables***	***Group***	***Pre***	***Post***	***Interaction (group × time)***	
				**F**	**P**
Civic attitudes (point)	Service-Learning	18.96 ± 2.74	24.75 ± 3.00	713.129	<0.001^***^
	Control	18.18 ± 2.72	18.12 ± 2.99		
Self-efficacy (point)	Service-Learning	105.49 ± 9.79	89.95 ± 5.23	465.731	<0.001^***^
	Control	100.40 ± 9.17	101.02 ± 9.17		

Data are presented as means ± standard deviations

Service-Learning program group, n=1,184; control group, n=298

*** *P*<0.001; tested by repeated measure analysis of variance

Thus, civic attitudes demonstrated positive improvement. However, the S-L participation group also demonstrated statistically decreased self-efficacy. Self-efficacy is normally increased by the performance of tasks regularly and through the successful completion of homework. Korean university students have, however, normally focused more on studying for college entrance examinations, this has led to a lack of experience in problem-solving in day-to-day life. Since S-L is their first social experience, they focus on the fact that they could not solve a problem rather than that they can solve a problem. Thus, one semester of S-L cannot affect successful experiences enough.

Nevertheless, S-L can be a meaningful learning opportunity for students to experience society and introspect. The strategy to encourage students to challenge their volunteer work and learning until they have enough successful experiences is necessary. In this regard, well-designed studies are imperative in the future.
